# Prophylaktische Strahlentherapie bei asymptomatischen Hochrisiko-Knochenmetastasen

**DOI:** 10.1007/s00066-023-02195-2

**Published:** 2024-01-05

**Authors:** Manuel Guhlich, Stefan Rieken

**Affiliations:** Klinik und Poliklinik für Strahlentherapie und Radioonkologie, Universitätsmedizin Göttingen, Robert-Koch-Str. 40, 37075 Göttingen, Deutschland

## Hintergrund

Knochenmetastasen (KM) stellen eine häufige Ursache für Morbidität und Mortalität bei Krebserkrankungen dar. Die palliative Radiotherapie (RT) ist bei schmerzhaft-symptomatischen Knochenmetastasen eine etablierte und effektive Therapie. KM verursachen nachgewiesenermaßen häufig (50–70 % in prospektiven Studien [2]) sogenannte skelettbezogene Ereignisse („skeletal related Events“, SRE). Als SRE werden pathologische Fraktur, Spinalkanalkompression, orthopädische Operationsindikation aufgrund Instabilität und analgetische RT definiert. Zur Prophylaxe von SRE werden üblicherweise osteoprotektive Medikamente (Bisphosphonate, Denosumab) eingesetzt. Retrospektive Daten konnten eine Assoziation zwischen RT und reduziertem SRE-Risiko nachweisen [3]. Prospektive Daten existierten bislang nicht.

## Patienten und Methodik

Zur Prüfung der Hypothese, dass RT bei asymptomatischen Hochrisiko-Knochenmetastasen (ahKM) die SRE-Rate reduziert, wurde eine multizentrisch randomisierte Phase-II-Studie durchgeführt. „Hochrisiko-Knochenmetastase“ war dabei definiert als Ausdehnung ≥ 2 cm, Beteiligung von Hüfte, Schulter oder Ileosacralgelenk, lange Röhrenknochen mit Befall von 1/3 bis 2/3 der Kortikalis, Wirbelkörper der Verbindungswirbelsäule (C7-T1, T12-L1, L5-S1) und/oder Hinterkantenbeteiligung. Als sekundäre Zielparameter wurden SRE-assoziierte stationäre Aufnahmen, Gesamtüberleben (overall survival, OS), schmerzassoziierte Lebensqualität und Therapietoxizität analysiert. Eingeschlossene Patienten (Pat.) waren multipel metastasiert (> 5 Metastasen) und hatten mindestens eine ahKM. Bereits erfolgte operative Stabilisierung sowie RT im betroffenen Areal waren Ausschlusskriterien. Pat. wurden 1:1 randomisiert: prophylaktische RT aller ahKM *vs.* Therapiestandard (Standart of Care, SOC). Das follow-up (FU) für den primären Endpunkt betrug 1 Jahr mit klinischen Visiten in Monat 3, 6 und 12 sowie zum Zeitpunkt einer SRE; bzgl. sekundärer Endpunkte darüber hinaus. Das Studienprotokoll wurde separat publiziert [4]. Insgesamt wurde 78 Pat. mit 122 ahKM eingeschlossen: 35 Pat mit 62 ahKM in den RT-Arm *vs*. 36 Pat. mit 49 ahKM in den SOC-Arm. Die Hälfte der Patienten erhielt eine ergänzende osteoprotektive Therapie.

## Ergebnisse

Im Rahmen des einjährigen Beobachtungszeitraums hinsichtlich des primären Endpunkts traten 15 SRE auf, davon 14 im SOC-Arm, 1 im RT-Arm (29 % vs. 1,6 %, *p* < 0,001). Dieses Ergebnis blieb auch nach Exklusion der häufigsten SRE (palliative RT bei Schmerzsymptomatik) statistisch signifikant (*p* = 0,008). Die hazard ratio für das Auftreten einer SRE betrug 0,09 (95 % CI: 0,01–0,66, *p* = 0,018) für den RT-Arm. SRE-bedingte stat. Aufnahmen gab es nach einem Jahr im RT-Arm nicht, wohingegen im SOC-Arm 4 Patienten stationär aufgenommen wurden (*p* = 0,045). Im SOC-Arm traten zudem 4 pathologische Frakturen und zwei Rückenmarkskompressionen auf. Das OS war bei einer medianen Nachbeobachtungszeit von 2,5 Jahren signifikant länger im RT-Arm (1,7 Jahre vs. 1 Jahr, *p* = 0,018), auch nach multivariabler Testung. Die therapieassoziierte Toxizität war gering ausgeprägt, Grad 3–5 Toxizitäten wurden nicht berichtet. Grad 2-Toxizitäten traten bei 13% der Patienten im RT-Arm vs. 3% im SOC-Arm auf.

## Schlussfolgerung der Autoren

Pat. mit ahKM profitieren von einer prophylaktischen RT durch eine signifikante Reduktion von SRE, einer Reduktion von Knochenschmerzen, konsekutiven stationären Aufenthalten sowie einem verlängerten OS. Eine Phase III-Studie zur Bestätigung der Ergebnisse an einem größeren Kollektiv sollte folgen.

## Kommentar

Die vorliegende Studie belegt eine herausragende Effektivität für die prophylaktische RT asymptomatischer Hochrisiko-Knochenmetastasen, welche deutlich über die etablierten RT-Indikationen (Schmerzen, Frakturgefahr) bei KM hinausgeht. Der klinische Benefit für die Patienten ist kaum deutlich genug zu betonen: Neben der eindrücklichen Risikoreduktion für SRE sind es auch und vor allem die konsekutiven (unter RT ausbleibenden) Folgen derer: Schmerzen, vor allem aber stationäre Aufenthalte, Operationen und die damit verbundenen Morbiditäts- und Mortalitätsrisiken, welche den Wert der RT in dieser Situation bedeuten. Auch wenn argumentiert werden kann, dass für einen Teil der Patienten eine spätere Bestrahlung ausreichend war, ist dem entgegenzuhalten, dass durch die frühzeitige RT pathologische Frakturen und Rückenmarkskompressionen im Studienzeitraum komplett verhindert werden konnten. Bedeutsam ist, dass die absolute Mehrheit der folgenden SRE innerhalb von 3 Monaten nach Studieneinschluss auftraten. Dieser Zeitraum ist in einem Kollektiv multipel metastasierter Pat. realistisch, was die klinische Relevanz neben der statistischen Signifikanz betont. Die nach Studienprotokoll durchgeführte RT entspricht den üblichen Standards auch der deutschen Radioonkologie: Das häufigste Fraktionierungsschema war 3 × 9 Gy, die überwiegende Mehrzahl der Metastasen wurden aber mit klassischen palliativen Schemata behandelt (5 × 4 Gy, 1 × 8 Gy und 10 × 3 Gy). Die therapieassoziierte Toxizität war (erwartungsgemäß) sehr moderat. Bemüht man den Vergleich von RT mit Metaanalysen für osteoprotektive Medikation, so zeigt sich eine deutlich bessere Wirkung der RT (z. B. Metaanalyse von Machado et al. [5]: HR zwischen 0,7–0,87 bzgl. SRE, kein Einfluss auf das OS). Auf Grund der geringen Fallzahl in der aktuellen Studie sollte die Gesamtüberlebensverbesserung weiterhin vorsichtig interpretiert werden. Die Kausalkette und darüber hinaus gezeigte Vorteile sind jedoch plausibel genug, um betroffenen Pat. bereits jetzt die Option der prophylaktischen RT anzubieten und die o. g. Argumentation selbstbewusst für die Radioonkologie und unsere Pat. im kollegialen Austausch zu vertreten. Dies gilt umso mehr für betroffene Bereiche wie Knochen der Verbindungswirbelsäule sowie bei Osteolysen ≥ 2 cm, welche die höchste Rate an SRE aufwiesen.

*Manuel Guhlich und Stefan Rieken, Göttingen*
